# Supplementary material for: Synthetic promoters to induce immune-effectors into the tumor microenvironment
Source: Commun Biol. 2021 Jan 29;4:143. doi: 10.1038/s42003-021-01664-7 (PMC7846768; doi:10.1038/s42003-021-01664-7)
Supplement: Supplementary file 2 — Supplementary Information [file 42003_2021_1664_MOESM2_ESM.pdf]

K1
GGGAATTTCCGGGGACTTTCCGGGAATTTCCGGGGACTTTCCGGGAATTTCCAGAGCATATTAAGGTGACGCGTGTGGCC
TCGAACACCGAGCGACCCTGCAGCGACCCGCTTAAAA
G2
TTCCGGGAAAGGGTGGGCAAGTTTCCGGGAAAGCAGTAGGTACAGCCTTCCGGGAAAGGGTGGGCAAGTTTCCGGGAAAG
CAGTAGGTTTTTCGCATATTAAGGTGACGCGTGTGGCCTCGAACACCGAGCGACCCTGCAGCGACCCGCTTAAAA
G4
TTCCGGGAAAGGGTGGGCAAGTTTCCGGGAAAGCAGTAGGTACAGCCTTCCGGGAAAGGGTGGGCAAGTTTCCGGGAAAG
CAGTAGGTACAGCCTTCCGGGAAAGGGTGGGCAAGTTTCCGGGAAAGCAGTAGGTACAGCCTTCCGGGAAAGGGTGGGCA
AGTTTCCGGGAAAGCAGTAGGTTTTTCGCATATTAAGGTGACGCGTGTGGCCTCGAACACCGAGCGACCCTGCAGCGACCC
GCTTAAAA
G6
TTTCCGGGAAAGGGTGGGCAAGTTTCCGGGAAAGCAGTAGGTACAGCCTTCCGGGAAAGGGTGGGCAAGTTTCCGGGAAA
GCAGTAGGTACAGCCTTCCGGGAAAGGGTGGGCAAGTTTCCGGGAAAGCAGTAGGTACAGCCTTCCGGGAAAGGGTGGGC
AAGTTTCCGGGAAAGCAGTAGGTACAGCCTTCCGGGAAAGGGTGGGCAAGTTTCCGGGAAAGCAGTAGGTACAGCCTTCC
GGGAAAGGGTGGGCAAGTTTCCGGGAAAGCAGTAGGTTTTTCGCATATTAAGGTGACGCGTGTGGCCTCGAACACCGAGC
GACCCTGCAGCGACCCGCTTAAAA
G1K06
TTCCGGGAAAGGGTGGGCAAGTTTCCGGGAAcccGGGAATTTCCGGGGACTTTCCGGGAATTTCCAGAGCATATTAAGGTGA
CGCGTGTGGCCTCGAACACCGAGCGACCCTGCAGCGACCCGCTTAAAA
G1K1
TTCCGGGAAAGGGTGGGCAAGTTTCCGGGAAcccGGGAATTTCCGGGGACTTTCCGGGAATTTCCGGGGACTTTCCGGGAAT
TTCCAGAGCATATTAAGGTGACGCGTGTGGCCTCGAACACCGAGCGACCCTGCAGCGACCCGCTTAAAA
G2K2
TTCCGGGAAAGGGTGGGCAAGTTTCCGGGAAAGCAGTAGGTACAGCCTTCCGGGAAAGGGTGGGCAAGTTTCCGGGAAAG
AGCAGGGAATTTCCGGGGACTTTCCGGGAATTTCCGGGGACTTTCCGGGAATTTCCAGAGCAGGGAATTTCCGGGGACTTTC
CGGGAATTTCCGGGGACTTTCCGGGAATTTCCAGAGCATATTAAGGTGACGCGTGTGGCCTCGAACACCGAGCGACCCTGC
AGCGACCCGCTTAAAA
G3K3
TTCCGGGAAAGGGTGGGCAAGTTTCCGGGAAAGCAGTAGGTACAGCCTTCCGGGAAAGGGTGGGCAAGTTTCCGGGAAAG
CAGTAGGTACAGCCTTCCGGGAAAGGGTGGGCAAGTTTCCGGGAAAGAGCAGGGAATTTCCGGGGACTTTCCGGGAATTTC
CGGGGACTTTCCGGGAATTTCCAGAGCAGGGAATTTCCGGGGACTTTCCGGGAATTTCCGGGGACTTTCCGGGAATTTCCAG
AGCAGGGAATTTCCGGGGACTTTCCGGGAATTTCCGGGGACTTTCCGGGAATTTCCAGAGCATATTAAGGTGACGCGTGTG
GCCTCGAACACCGAGCGACCCTGCAGCGACCCGCTTAAAA
G1K06H1
TTCCGGGAAAGGGTGGGCAAGTTTCCGGGAAcccGGGAATTTCCGGGGACTTTCCGGGAATTTCCtacaGACCTTGAGTACGT
GCGTCTCTGCACGTATGAGAGCATATTAAGGTGACGCGTGTGGCCTCGAACACCGAGCGACCCTGCAGCGACCCGCTTAAA
A
H2G2K2
GACCTTGAGTACGTGCGTCTCTGCACGTATGAGAGCAGACCTTGAGTACGTGCGTCTCTGCACGTATGAGAGCATTCCGGGA
AAGGGTGGGCAAGTTTCCGGGAAAGCAGTAGGTACAGCCTTCCGGGAAAGGGTGGGCAAGTTTCCGGGAAAGAGCAGGG
AATTTCCGGGGACTTTCCGGGAATTTCCGGGGACTTTCCGGGAATTTCCAGAGCAGGGAATTTCCGGGGACTTTCCGGGAAT
TTCCGGGGACTTTCCGGGAATTTCCAGAGCATATTAAGGTGACGCGTGTGGCCTCGAACACCGAGCGACCCTGCAGCGACCC
GCTTAAAA
G2H2K2
TTCCGGGAAAGGGTGGGCAAGTTTCCGGGAAAGCAGTAGGTACAGCCTTCCGGGAAAGGGTGGGCAAGTTTCCGGGAAAG
CAGTAGGTACAGCCGACCTTGAGTACGTGCGTCTCTGCACGTATGAGAGCAGACCTTGAGTACGTGCGTCTCTGCACGTATG
AGAGCAGGGAATTTCCGGGGACTTTCCGGGAATTTCCGGGGACTTTCCGGGAATTTCCAGAGCAGGGAATTTCCGGGGACT
TTCCGGGAATTTCCGGGGACTTTCCGGGAATTTCCAGAGCATATTAAGGTGACGCGTGTGGCCTCGAACACCGAGCGACCCT
GCAGCGACCCGCTTAAAA
G2K2H2
TTCCGGGAAAGGGTGGGCAAGTTTCCGGGAAAGCAGTAGGTACAGCCTTCCGGGAAAGGGTGGGCAAGTTTCCGGGAAAG
AGCAGGGAATTTCCGGGGACTTTCCGGGAATTTCCGGGGACTTTCCGGGAATTTCCAGAGCAGGGAATTTCCGGGGACTTTC
CGGGAATTTCCGGGGACTTTCCGGGAATTTCCAGAGCAGACCTTGAGTACGTGCGTCTCTGCACGTATGAGAGCAGACCTTG
AGTACGTGCGTCTCTGCACGTATGAGAGCATATTAAGGTGACGCGTGTGGCCTCGAACACCGAGCGACCCTGCAGCGACCC
GCTTAAAA
Herceptin based CAR
Atgatggattttcaggtgcagattttcagcttcctgctaatcagtgcctcagtcataatgtccagaggagatatccagatgacccagtccccgagctccctgtccgcct
ctgtgggcgatagggtcaccatcacctgccgtgccagtcaggatgtgaatactgctgtagcctggtatcaacagaaaccaggaaaagctccgaaactactgattta
ctcggcatccttcctttattctggagtcccttctcgcttctctggatctagatctgggacggatttcactctgaccatcagcagtctgcagccggaagacttcgcaactt
attactgtcagcaacattatactactcctcccacgttcggacagggtaccaaggtggagatcaaacgcactgggtctacatctggatctgggaagccgggttctggt
gagggttctgaggttcagctggtggagtctggcggtggcctggtgcagccaggggctcactccgtttgtcctgtgcagcttctggcttcaacattaaagacacctat
atacactgggtgcgtcaggccccgggtaagggcctggaatgggttgcaaggatttatcctacgaatggttatactagatatgccgatagcgtcaagggccgtttcac

tataagcgcagacacatccaaaaacacagcctacctgcagatgaacagcctgcgtgctgaggacactgccgtctattattgttctagatggggaggggacggcttc
tatgctatggacgtgtggggtcaaggaaccctggtcaccgtctcctcgctcgaggaacaaaaactcatctcagaagaggatctgttcgtgccggtcttcctgccagc
gaagcccaccacgacgccagcgccgcgaccaccaacaccggcgcccaccatcgcgtcgcagcccctgtccctgcgcccagaggcgtgccggccagcggcggggg
gcgcagtgcacacgagggggctggacttcgcctgtgatatctacatctgggcgcccttggccgggacttgtggggtccttctcctgtcactggttatcaccctttactg
caaccacaggaacaggagtaagaggagcaggctcctgcacagtgactacatgaacatgactccccgccgccccgggcccacccgcaagcattaccagccctatg
ccccaccacgcgacttcgcagcctatcgctcccgtttctctgttgttaaacggggcagaaagaagctcctgtatatattcaaacaaccatttatgagaccagtacaaa
ctactcaagaggaagatggctgtagctgccgatttccagaagaagaagaaggaggatgtgaactgagagtgaagttcagcaggagcgcagacgcccccgcgtac
cagcagggccagaaccagctctataacgagctcaatctaggacgaagagaggagtacgatgttttggacaagagacgtggccgggaccctgagatggggggaa
agccgagaaggaagaaccctcaggaaggcctgtacaatgaactgcagaaagataagatggcggaggcctacagtgagattgggatgaaaggcgagcgccgga
ggggcaaggggcacgatggcctttaccagggtctcagtacagccaccaaggacacctacgacgcccttcacatgcaggccctgccccctcgctaa

Supplementary Figure 1 – The full CARTIV promotors sequences and CAR used: turquois – KCPRE element, yellow- GCPRE element, green – HCPRE element, pink – TATA box

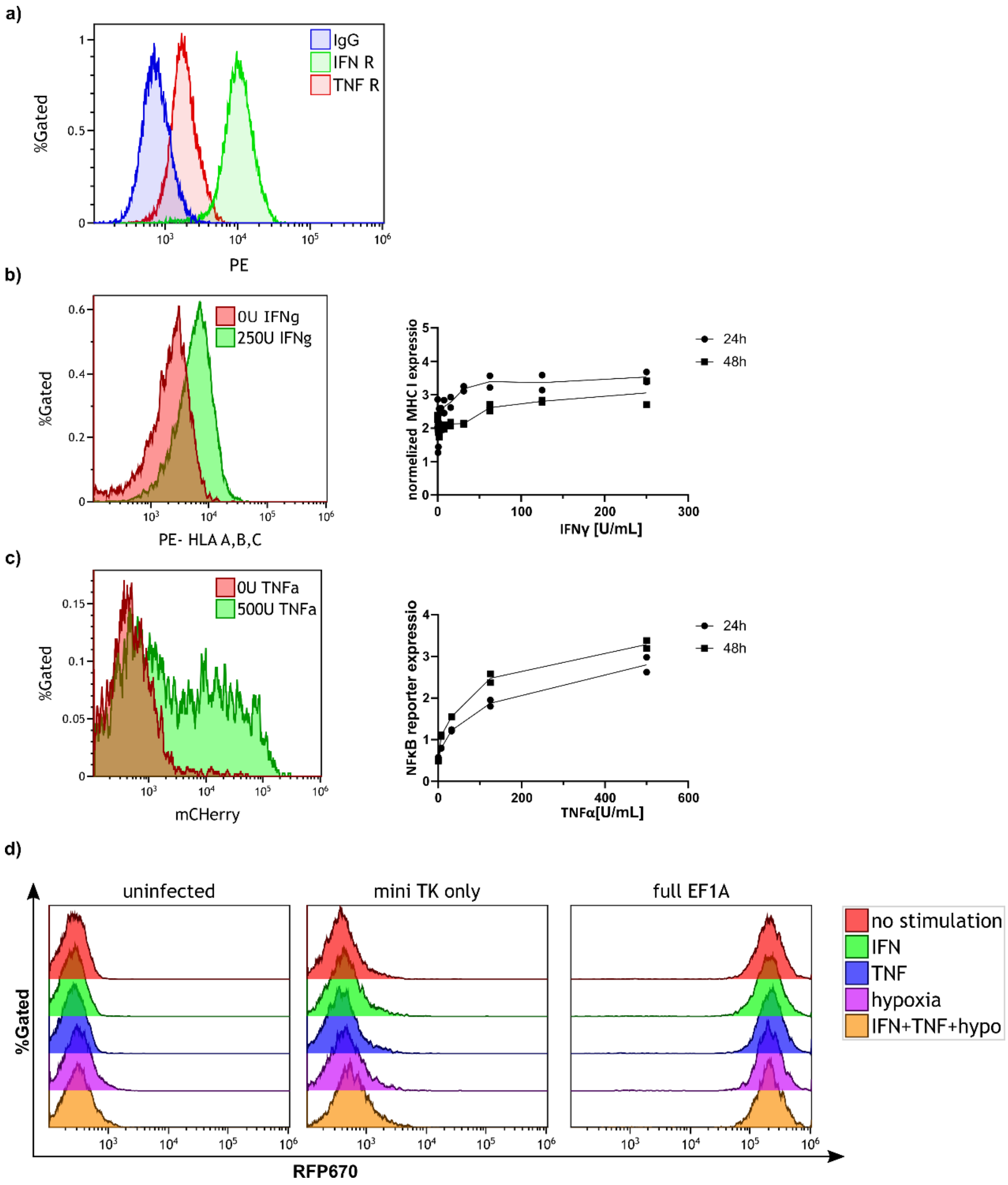

Supplementary Figure 2 - HEK293T cells are responsive to IFNγ and TNFa: a. representative FACS staining of HEK293T cells for IFNγ and TNFa receptors b. left panel - representative FACS staining of HEK293T stained with anti HLA-A,B,C -PE (clone W6/32) after incubation with 250U/mL of IFNγ for 24 and 48 hours. right panel - representative Dose response of HEK293T cells to IFNγ. data is single discriminated and DAPI negative. Showing average gMEAN of duplicates, error bars indicate standard deviation. c. left panel - representative FACS plot of HEK293 cells co transfected with N1 GFP and NFkB mCherry reporter plasmid. Data shown is GFP positive, single discriminated and DAPI negative. right panel - representative Dose response of HEK293T to TNFα. 24 hours after transfection cells were harvested and plated again to be incubated with the indicated concentration of TNFα. data is single discriminated ,GFP positive and DAPI negative, data shown is an average of duplicates of gMEAN of reporter expression, error bar indicate standard deviation. d. representative FACS plot of control constructs. left panel - uninfected HEK293 cells; middle panel- HEK293 infected using lentiviral vectors with RFP670 under the control of minimal TK promotor only and ZsGreen controlled by the ef1α core promotor. right panel - HEK293 cells infected using lentiviral vectors with RFP670 under the control of full EF1A promotor and ZsGreen controlled by the ef1α core promotor. At 72 hours following infection, the cells were incubated for 48 hours with the indicated cytokines (500 U/mL for each cytokine) and

placed under hypoxic conditions for 24 hours, harvested and analyzed by flow cytometry. Data shown are ZsGreen-positive, single-discriminated and DAPI-negative. Results are from one representative experiment of two performed.

a)

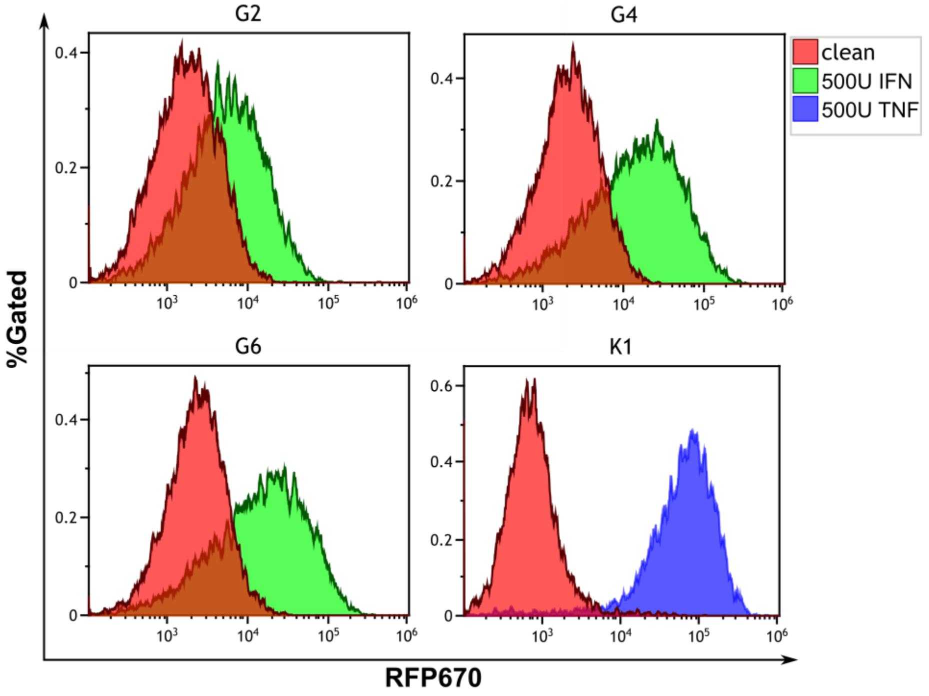

b)

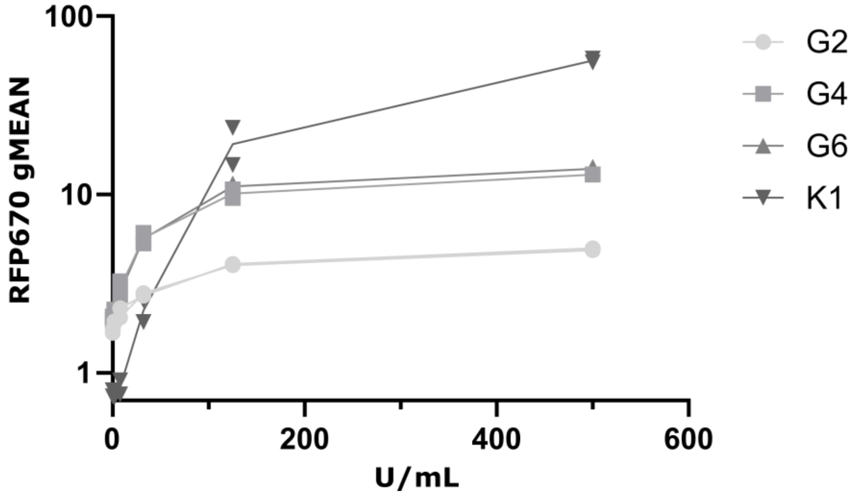

Supplementary figure 3 – single CPRE promotors are induced by IFNγ and TNFα stimulation in HEK293 cells in a dose response manner: a. Representative plots of HEK293T cells infected using lentiviral vectors with RFP670 under the control of the indicated CARTIV promotor and ZsGreen controlled by the ef1α core promotor. At 72 hours following infection, the cells were incubated for 48 hours with the indicated cytokines (500 U/mL for each cytokine), harvested and analyzed by flow cytometry. Data shown are ZsGreen-positive, single-discriminated and DAPI-negative results b. Geometric mean of RFP670 in ZsGreen-positive cells, showing average of duplicates; error bars indicate standard deviation. Results are from one representative experiment of two performed.

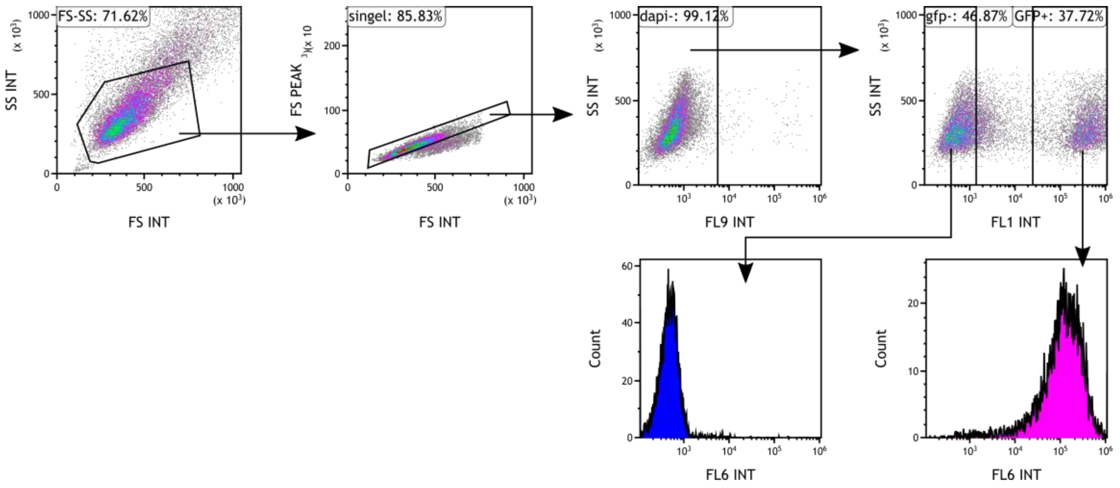

Supplementary Figure 4 – Gating strategy for assessing the CARTIV promotor response to cytokines: The gating strategy used for evaluating direct activation of CARTIV vectors. Representative plot of HEK293T cells infected using a lentiviral vector with RFP670 under the control of the G1K1 CARTIV promotor and ZsGreen controlled by the ef1α core promotor.

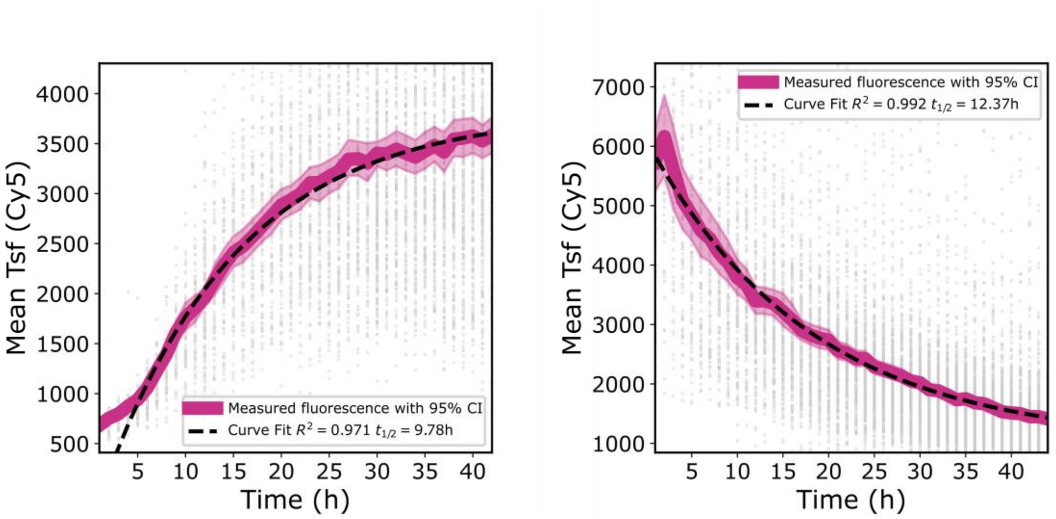

Supplementary Figure 5 – kinetics of the G1K06H1 promotor: HEK293T cells infected using lentiviral vectors with RFP670 under the control of the G1K06H1 CARTIV promotor and ZsGreen controlled by the ef1α core promotor were either stimulated using IFNγ and TNFα 500U/mL for 48 hours and then washed twice in complete DMEM or supplemented with IFNγ and TNFα 500U/mL before starting the measurement. Saturation curve: Gray points represent the raw measurements. The solid pink line represents the mean for every time point. Transparent borders around the mean represent a 95% CI range. The black dashed line represents a reverse exponential curve fitted to the data. Decay curve: Gray points represent the raw measurements. The solid pink line represents the mean for every time point. Transparent borders around the mean represent a 95% CI range. The black dashed line represents an exponential decay curve fitted to the data.

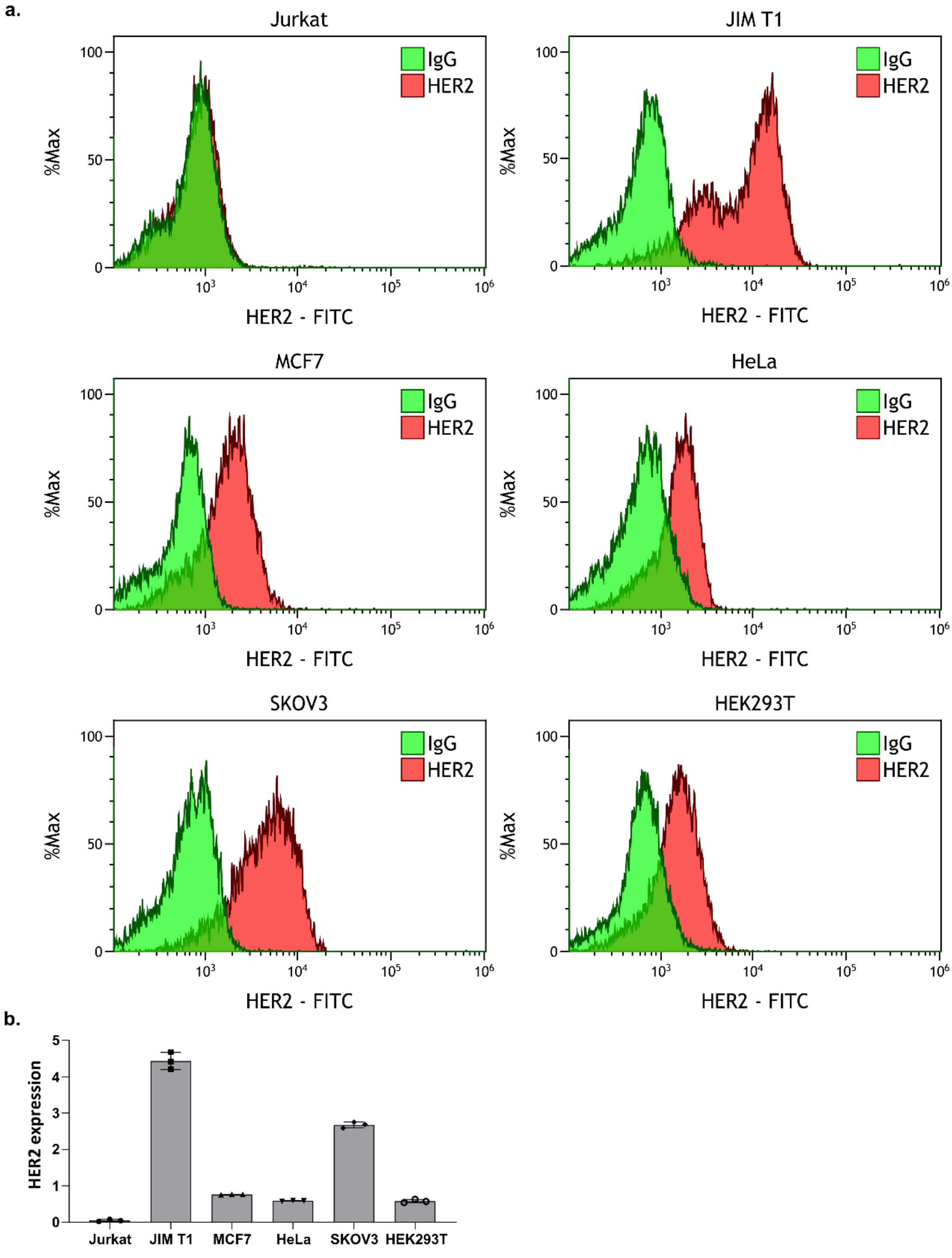

Supplementary Figure 6 - The selected target cell lines used to test the Herceptin based CAR and gating for calculation of CD107a positive cells: a. representative HER2 staining for all the cell lines used to test the Herceptin based car under the control of the CARTIV promotor. Cells were harvested using versin and stained using an isotype control or HER2-FITC and analyzed by flow cytometry. Data shown are single-discriminated and DAPI-negative b. HER2 expression of all the cell used to test the Herceptin based CAR. expression was calculated by the formula : (gMEAN HER2 staining)- (gMEAN isotype control). Showing average of triplicates, error bars indicate standard deviation. Results for 6A are from one representative experiment of three performed.

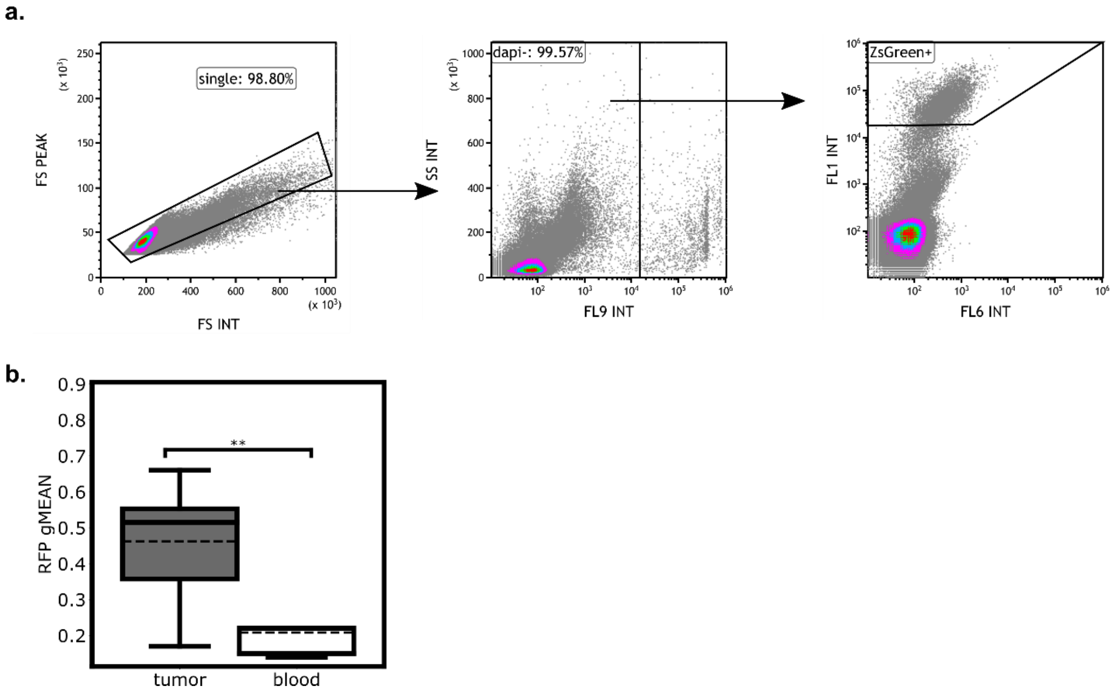

Supplementary Figure 7 – Gating strategy for assessing the CARTIV promotor response in vivo: a. the gating strategy used for the NK92 G1K06H1 CARTIV vector in vivo. Representative plots of NK92 cells infected with lentiviral vectors with RFP670 under the control of the G1K06H1 CARTIV promotor and ZsGreen controlled by the ef1α core promotor. Tumors and Matrigel plugs were dissociated and analyzed by FACS b. Summary of reporter expression in the CDX compared with the blood; number of tested tumors is ten. Box borders represent upper and lower quartiles. Solid lines represent the median and dashed line the mean. Whiskers represent maximum and minimum. *, P-value < 0.05; **, P-value < 0.005; ***, P-value < 0.0005.
