# Supplementary material for: Synthetic promoters to induce immune-effectors into the tumor microenvironment
Source: Commun Biol. 2021 Jan 29;4:143. doi: 10.1038/s42003-021-01664-7 (PMC7846768; doi:10.1038/s42003-021-01664-7)
Supplement: Supplementary file 3 — Description of Additional Supplementary Files [file 42003_2021_1664_MOESM3_ESM.pdf]

Description of Additional Supplementary Files

Name: Supplementary Data 1

Description: Source data of the figures presented in manuscript “Synthetic promoters to induce immune effectors in the tumor microenvironment” by Greenshpan et al.
